# Bilateral Amygdala Radio-Frequency Ablation for Refractory Aggressive Behavior Alters Local Cortical Thickness to a Pattern Found in Non-refractory Patients

**DOI:** 10.3389/fnhum.2021.653631

**Published:** 2021-06-09

**Authors:** Flavia Venetucci Gouveia, Jürgen Germann, Gabriel A. Devenyi, Erich T. Fonoff, Rosa M. C. B. Morais, Helena Brentani, M. Mallar Chakravarty, Raquel C. R. Martinez

**Affiliations:** ^1^Biological Sciences Platform, Sunnybrook Research Institute, Toronto, ON, Canada; ^2^Division of Neuroscience, Sirio-Libanês Hospital, São Paulo, Brazil; ^3^Division of Neurosurgery, Department of Surgery, University Health Network and University of Toronto, Toronto, ON, Canada; ^4^Cerebral Imaging Centre, Douglas Mental Health University Institute, McGill University, Montreal, QC, Canada; ^5^Department of Psychiatry, McGill University, Montreal, QC, Canada; ^6^Department of Neurology, Division of Functional Neurosurgery of the Institute of Psychiatry, University of São Paulo, Medical School, São Paulo, Brazil; ^7^LIM/23, Department of Psychiatry, University of São Paulo, Medical School, São Paulo, Brazil; ^8^Department of Biological and Biomedical Engineering, McGill University, Montreal, QC, Canada

**Keywords:** aggressive behavior, amygdala, cortical thickness (CT), magnetic resonance imaging, neurosurgery, self-injury behavior

## Abstract

Aggressive behaviors comprise verbal and/or physical aggression directed toward oneself, others, or objects and are highly prevalent among psychiatric patients, especially patients diagnosed with autism spectrum disorder and severe intellectual disabilities. Some of these patients are considered refractory to treatment, and functional neurosurgery targeting the amygdala can result in widespread plastic brain changes that might reflect ceasing of some abnormal brain function, offering symptom alleviation. This study investigated cortical thickness changes in refractory aggressive behavior patients that were treated with bilateral amygdala ablation and compared to control patients presenting non-refractory aggressive behavior [three refractory and seven non-refractory patients, all males diagnosed with autism spectrum disorder (ASD) and intellectual disabilities]. The Overt Aggression Scale (OAS) was used to quantify behavior and magnetic resonance imaging was performed to investigate cortical thickness. Before surgery, both groups presented similar total OAS score, however refractory patients presented higher physical aggression against others. After surgery the refractory group showed 88% average reduction of aggressive behavior. Imaging analysis showed that while refractory patients present an overall reduction in cortical thickness compared to non-refractory patients across both timepoints, the local pattern of thickness difference found in areas of the neurocircuitry of aggressive behavior present before surgery is diminished and no longer detected after surgery. These results corroborate the hypotheses on induction of widespread neuronal plasticity following functional neurosurgical procedures resulting in modifications in brain morphology and improvement in behavior. Further studies are necessary to determine the underlying cause of these morphological changes and to better understand and improve treatment options.

## Introduction

Aggressive behavior is a multifactorial behavioral syndrome that includes physical aggression and/or verbal aggression and can be directed at oneself (self-injury behavior), to others, and to objects (Corrigan et al., 1993; Yudofsky et al., 2012). It has a high prevalence in patients with Autism Spectrum Disorder and in patients with intellectual disabilities, being a leading cause for patient institutionalization (Brentani et al., 2013; Adler et al., 2015; Gouveia et al., 2019, 2020). Pharmacological therapies targeting the serotonergic and dopaminergic systems (e.g., typical and atypical antipsychotics, as well as selective serotonin reuptake inhibitors), anti-epileptic agents (e.g., valproic acid and carbamazepine), psychostimulants (e.g., amphetamine, methylphenidate), and a large variety of mood stabilizers (e.g., lithium) and tranquilizers (e.g., benzodiazepines) are commonly used to treat aggressive behavior in association with behavioral therapy (Morrissette and Stahl, 2014; Adler et al., 2015; Coleman et al., 2019). Alternative high dose mono and poly-pharmacy therapies can be offered when patients fail to present behavioral improvement with standard therapy (Morrissette and Stahl, 2014). However, these alternative therapies have been associated with symptom deterioration and their long-term side effects are poorly understood. Patients whose behavior does not improve after exhausting all available medication therapies are considered to have refractory aggressive behavior (Adler et al., 2015; Gouveia et al., 2021) and functional neurosurgical procedures may be considered to alleviate symptoms (Sturm et al., 2012; Gouveia et al., 2019, 2021).

The amygdala is a central structure of the neurocircuitry of aggressive behavior that also includes cortical areas (e.g., the orbitofrontal cortex, cingulate cortex) and subcortical structures (e.g., hypothalamus, nucleus accumbens, periaqueductal gray matter) (Siever, 2008; Blair, 2016; Gouveia et al., 2019). An imbalance in serotonin transmission in prefrontal cortical regions along with an increase in limbic activity (especially the amygdala) is believed to be the underlying cause for excessive aggressive behavior (Siever, 2008; Blair, 2016; Gouveia et al., 2019). Previous studies have shown that the bilateral ablation of the amygdala in patients with a wide range of psychiatric disorders associated with severe and/or refractory aggressive behavior can successfully reduce the frequency and/or severity of aggressive behavior and improve patients’ quality of life (Kiloh et al., 1974; Balasubramaniam and Kanaka, 1975; Gouveia et al., 2019, 2021). The marked reduction in aggressive behavior observed in these severe cases following the ablation of the amygdala will affect the neurocircuitry of aggressive behavior, and this circuit-wide restoration of functional equilibrium should be associated with measurable plastic changes (Siever, 2008). To address this hypothesis, in this study we investigated changes in cortical thickness in patients presenting refractory aggressive behavior who were treated with bilateral amygdala radio-frequency ablation for symptom alleviation as compared to patients presenting non-refractory aggressive behavior.

## Materials and Methods

### Subjects

A total of 10 patients with severe aggressive behavior hallmarked by severe self-injury behavior and extreme aggression toward objects and others were included in this study. All patients were males diagnosed with autism spectrum disorder (ASD) and severe intellectual disability, as qualitatively described by the Diagnostic and Statistical Manual of Mental Disorders, 5th Edition (DSM-5; American Psychiatric Association [APA], 2013). Tests of intelligence quotient (IQ) were not performed as patients were not collaborative, presented high motor agitation and had limited ability to communicate. Of those, three patients were considered to have refractory aggressive behavior (Gouveia et al., 2020, 2021), defined as persistent aggressive behavior despite previous trials of FDA approved antipsychotics targeting the core symptom (Adler et al., 2015), previous trials of single and poly-medication (including antidepressants, mood stabilizers, psycho-stimulants and anticonvulsants) (Morrissette and Stahl, 2014; Gouveia et al., 2019) and insufficient improvement following behavioral therapy. Refractory patients were treated with bilateral amygdala radio-frequency ablation to reduce aggressive behavior (for full description of the surgical procedure see Gouveia et al., 2021). The remaining seven patients were considered non-refractory, as they did not exhaust all possible medication therapies, and surgery was not considered for these patients. Table 1 provides detailed demographics. All procedures were approved by the Research Ethics Board (CAPPesq #742.331; CAAE #31828014.6.3001.5461), and written informed consent was obtained from the patients’ parents.

**TABLE 1:** Demographics and clinical characteristics.

**Case**	**Medication history**		**Age**	**CARS**	**Overt Aggression Scale**				
					**Total**	**Verbal**	**Objects**	**Self**	**Others**
1	Biperiden (2 mg)	Olanzapine (5 mg)	19	55					
	Carbamazepine (200 mg)	Promethazine (25 mg)							
	Chlorpromazine (100 mg)	Quetiapine fumarate (100 mg)			Pre:13	Pre:1	Pre:4	Pre:4	Pre:4
	Divalproex sodium (500 mg)	Risperidone (2 mg)			Post:2	Post:0	Post:0	Post:1	Post:1
	Haloperidol (5 mg)	Thioridazine (200 mg)			IR = 0.9	IR = 1	IR = 1	IR = 0.8	IR = 0.8
	Levomepromazine (100 mg)	Ziprasidone (80 mg)							
2	Aripiprazole (15 mg)	Haloperidol (5 mg)	27	53					
	Biperiden (2 mg)	Levomepromazine (100 mg)							
	Carbamazepine (200 mg)	Olanzapine (5 mg)			Pre:10	Pre:1	Pre:4	Pre:1	Pre:4
	Chlorpromazine (100 mg)	Promethazine (25 mg)			Post:2	Post:1	Post:1	Post:0	Post:1
	Clobazam (10 mg)	Risperidone (2 mg)			IR = 0.8	IR = 1	IR = 0.8	IR = 1	IR = 0.8
	Clonidine (0.1 mg)	Thioridazine (200 mg)							
	Clozapine (100 mg)	Topiramate (50 mg)							
	Divalproex sodium (500 mg)	Ziprasidone (80 mg)							
3	Aripiprazole (15 mg)	Oxcarbazepine (300 mg)	29	48					
	Biperiden (2 mg)	Promethazine (25 mg)							
	Carbamazepine (200 mg)	Quetiapine fumarate (50 mg)							
	Chlorpromazine (100 mg)	Risperidone (2 mg)			Pre:12	Pre:1	Pre:3	Pre:4	Pre:4
	Clomipramine (50 mg)	Sertraline (150 mg)			Post:0	Post:0	Post:0	Post:0	Post:0
	Clonazepam (2 mg)	Thioridazine (200 mg)			IR = 1	IR = 1	IR = 1	IR = 1	IR = 1
	Divalproex sodium (500 mg)	Topiramate (50 mg)							
	Haloperidol (5 mg)	Ziprasidone (80 mg)							
	Levomepromazine (100 mg)								
	Olanzapine (10 mg)								
4	Clobazam (10 mg)	Lithium (300 mg)	20	54	10	1	3	3	3
	Divalproex sodium (500 mg) Levomepromazine (100 mg)	Risperidone (1 mg)							
		Topiramate (100 mg)							
5	Carbamazepine (200 mg)	Promethazine (25 mg)	21	47	9	1	3	2	2
	Divalproex sodium (500 mg)	Quetiapine fumarate (100 mg)							
	Lithium (300 mg)	Risperidone (2 mg)							
6	Clobazam (10 mg)	Clozapine (100 mg)	19	51	11	0	4	3	4
	Clonidine (0.1 mg)	Lamotrigine (25 mg)							
7	Biperiden (2 mg)	Haloperidol (6 mg)	11	48	7	1	4	0	2
	Divalproex sodium (250 mg)								
8	Biperiden (2 mg)	Lithium (300 mg)	24	54	12	1	4	4	3
	Divalproex sodium (250 mg)	Olanzapine (10 mg)							
	Levomepromazine (100 mg)								
9	Carbamazepine (200 mg)	Levomepromazine (25 mg)	13	53	9	1	3	1	4
	Haloperidol (5 mg)								
10	Levomepromazine (25 mg)	Phenobarbital (50 mg)	20	54	7	1	1	3	2
	Nitrazepam (5 mg)	Risperidone (1 mg)							

*CARS, Childhood Autism Rating Scale; OAS, Overt Aggression Scale; IR, Improvement Rate.*

*Age given in years.*

### Questionnaire

The Overt Aggression Scale (OAS) was used to quantify aggressive behavior. The scale is divided in four domains to evaluate all aspects of the behavior and scored from 0 to 16, with 0 representing absence of the behavior and 4 representing the most extreme behavior for each domain (verbal aggression, physical aggression against objects, physical aggression against oneself, and physical aggression against other people) (Silver and Yudofsky, 1991; Yudofsky et al., 2012). No neuropsychological assessments were performed as patients presented with severe intellectual disability and were non-verbal. All questionnaires were reported by the patients’ parents and were performed concomitant with imaging magnetic resonance imaging (MRI) acquisition.

### Magnetic Resonance Imaging Acquisition and Processing

Magnetic resonance imaging was performed with patients under deep sedation using a 1.5 Tesla MRI system (Magnetom Espree, Siemens, Germany). T1-weighted structural images were acquired with the following parameters: slice thickness 1.0 mm, no gap, voxel size 1 mm × 0.9 mm × 0.9 mm or 1 mm × 0.5 mm × 0.5 mm, TE/TR 5/300 ms, flip angle 45°, FOV 240 mm. For patients with refractory aggressive behavior, MRI was performed before (on the week preceding surgery for surgical planning) and after bilateral radio-frequency amygdala ablation (3 months for case 2 and 12 months for cases 1 and 3; Supplementary Figure 1B). Non-refractory patients were scanned once. Imaging acquisitions was restricted to the minimum as the cases included here present a higher risk of respiratory depression and death during sedation (Gouveia et al., 2020, 2021). Images were processed as previously described (Bedford et al., 2020; Gouveia et al., 2020). Briefly, images were non-uniformity corrected using the iterativeN4 from minc-bpipe-library^1^ and subsequently processed using the CIVET pipeline (v2.1.0).^2^ Images were non-linearly registered to MNI152, and tissue classification was performed using classification priors (gray and white matter and cerebrospinal fluid). Cortical thickness was computed for all patients, at each vertex based on the distance between gray and white matter surfaces using Laplace’s method and blurred with a 40 mm geodesic surface kernel. Quality control of CIVET outputs was performed for all patients. Supplementary Figure 1A shows examples of the CIVET-based segmentations for refractory and non-refractory patients.

### Statistical Analysis

For cortical thickness analysis the RMINC (v.1.5.2.0)^3^ package in R (v.3.4.4)^4^ was used. The questionnaires’ measures were analyzed using Student’s *t*-test. Brain wide differences in cortical thickness were evaluated comparing refractory versus non-refractory patients at two timepoints: I. refractory before surgery versus controls and II. refractory after surgery versus controls. To this aim, linear models of thickness by group using individual mean cortical thickness as the covariate to correct for global differences were computed at each vertex. All analyses were corrected for multiple comparisons using False Discovery Rate (FDR) at the *p*_*FDRcor*_ < 0.05 threshold.

## Results

Only male patients were included in this study. Ages did not differ between patients with refractory (25.66 ± 4.50 years) and non-refractory (18.28 ± 4.60 years) aggressive behavior (*t* = 1.91, df = 3.39, *p* = 0.1415). No patient developed post-operative hydrocephalus in the 12-month follow-up and, aside from surgical scars and amygdala lesions, brain morphology and the MRI signal were considered normal and no alterations were detected in relation to the preoperative MRI. Before surgery, no significant differences were found between refractory and non-refractory patients in total OAS score or in the subscales of verbal aggression, physical aggression against objects, and physical aggression against self; however, refractory patients presented significantly higher levels of physical aggression against other people (Table 2). After surgery the refractory group showed a marked reduction in aggressive behavior, showing reduction in total OAS score (88% average) and in all subscales: verbal aggression (66% average), physical aggression against objects (91% average), physical aggression against oneself or himself (91% average), and physical aggression against other people (91% average; see Table 1 for individual improvement rate). Comparing refractory patients after surgery to the non-refractory patients, we observed significant differences in total OAS score and in the subscales of physical aggression against objects, physical aggression against self, and physical aggression against other people (Table 2). No significant difference was found in verbal aggression (Table 2).

**TABLE 2:** Overt Aggression Scale (OAS) score.

**Scale**	**Group**	**Mean ± SD**	***t*-value**	**df**	***p*-value**
**Refractory before surgery versus non-refractory**					
Total-OAS	Refractory	11.67 ± 1.53	2.098	4.7965	0.09235
	Non-refractory	9.29 ± 1.89			
Verbal Aggression	Refractory	1.00 ± 0.00	1	6	0.3559
	Non-refractory	0.86 ± 0.38			
Physical Aggression Against Objects	Refractory	3.67 ± 0.58	1	7.0918	0.3502
	Non-refractory	3.14 ± 1.07			
Physical Aggression Against Oneself	Refractory	3.00 ± 1.73	0.6333	3.1586	0.5694
	Non-refractory	2.29 ± 1.38			
Physical Aggression Against Other People	Refractory	4.00 ± 0.00	3.3607	6	0.01522 *
	Non-refractory	2.89 ± 0.90			
**Refractory after surgery versus non-refractory**					
Total-OAS	Refractory	1.33 ± 1.15	−8.1391	6.4112	0.00013***
	Non-refractory	9.29 ± 1.89			
Verbal Aggression	Refractory	0.33 ± 0.58	−1.4444	2.771	0.2515
	Non-refractory	0.86 ± 0.38			
Physical Aggression Against Objects	Refractory	0.33 ± 0.58	−5.3636	7.0918	0.001005**
	Non-refractory	3.14 ± 1.07			
Physical Aggression Against Oneself	Refractory	0.33 ± 0.58	−3.1538	7.9325	0.01367*
	Non-refractory	2.29 ± 1.38			
Physical Aggression Against Other People	Refractory	0.33 ± 0.58	−5.3	6.12	0.00172**
	Non-refractory	2.89 ± 0.9			

Levels of significance: *p < 0.05; **p < 0.01; ***p < 0.001.

**FIGURE 1:**
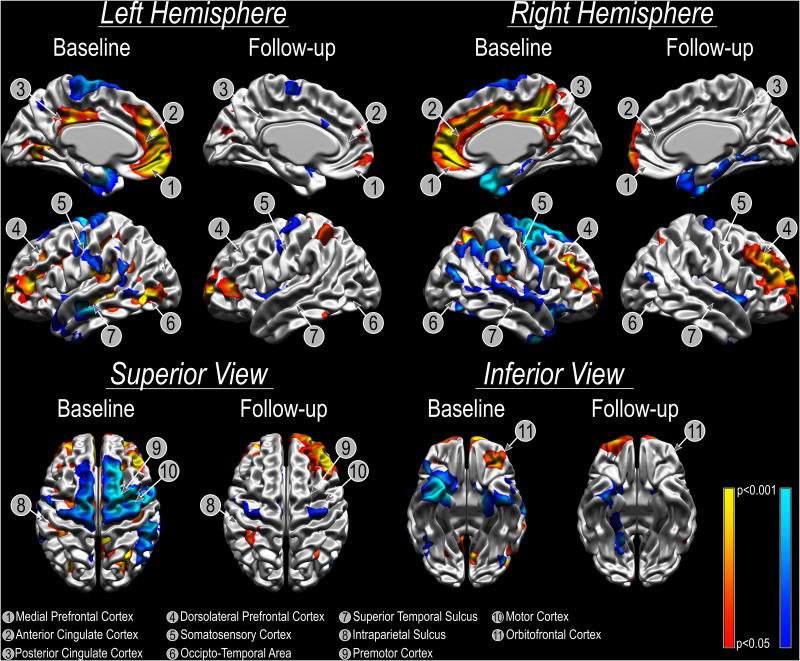
Cortical thickness analysis using CIVET. Cold colors show areas where CT is reduced in patients with refractory aggressive behavior compared with patients with non-refractory aggressive behavior. Warm colors show local cortical thickness increases comparing patients with refractory aggressive behavior to patients with non-refractory aggressive behavior. Please note that there were no significant differences between groups at follow up and colors in the images are uncorrected for multiple comparisons to better illustrate the extent and spatial pattern of the cortical thickness results.

Refractory patients presented an overall reduced cortical thickness compared to non-refractory subjects and the bilateral amygdala ablation did not change this morphological characteristic [Before surgery: left (*t* = −5.42, *p* < 0.001) and right (*t* = −5.61, *p* < 0.001) hemispheres; after surgery: left (*t* = −8.17, *p* < 0.001) and right (*t* = −4.76, *p* = 0.001) hemispheres). However, the vertex-wise relative cortical thickness analysis identified areas that showed significantly different cortical thickness (both increased/decreased) in refractory patients before surgery when compared to non-refractory patients (*p*_*FDRcor*_ < 0.05; Figure 1). Local bilateral increases in cortical thickness were found in the anterior cingulate cortex and posterior cingulate cortex, medial prefrontal cortex, dorsolateral prefrontal cortex, intraparietal sulcus, and orbitofrontal cortex (Figure 1). Local bilateral reduction in cortical thickness was observed in the somatosensory cortex, superior temporal sulcus, premotor cortex, and motor cortex (Figure 1). The local differences diminish following surgery as no significant differences can any longer be found between refractory and non-refractory subjects (Figure 1). While the local thickness differences in the right dorsolateral prefrontal cortex seem to be an exception to that general trend, the fact that no vertex survives multiple comparison corrections suggests that these results may be spurious.

## Discussion

This study has investigated changes of cortical thickness and a number of different aggressive behaviors following treatment in patients with refractory aggressive behavior compared to non-refractory patients. Both groups presented similar levels of total aggressive behavior at baseline and after bilateral amygdala radio-frequency ablation the refractory group showed a marked reduction of aggressive behavior now significantly different from the non-refractory group in total OAS and all subscales except for verbal aggression. However, it should be noted that since all patients included in this study present severe intellectual disability and are non-verbal, the range of this subscale is markedly limited with possible scores 0 (absent) or 1 (makes loud noises/shouts angrily). All refractory patients showed score 1 before surgery and after the procedure only one continued with this behavior, with the other two patients scoring zero. Interestingly, this patient that maintained verbal aggressive behavior after surgery (i.e., case 2) presented an average lesion size of 50% of the total volume of the amygdala (reaching parts of the lateral, basal, and central nuclei), while the remaining patients presented almost complete amygdala ablation. The amygdala is a key structure of the limbic system responsible for integrating concurrent inputs from the sensory systems and projecting this information to distinct brain regions to regulate emotional responses. The lateral nucleus of the amygdala in the main area receiving and processing these inputs and along with the basal nucleus, projects highly integrated multimodal information to the central nucleus, the most prominent output area. The high average improvement of the refractory group following amygdala ablation is in line with previous studies showing high rates of success after functional neurosurgery of the amygdala for aggressive behavior (Lee et al., 1998; Langevin et al., 2016; Gouveia et al., 2019, 2021). It is believed that a reduced top-down inhibitory control of the prefrontal cortex over the amygdala would result in hyper-activation of this nucleus and consequent activation of hypothalamic and brainstem regions resulting in lower tolerance of stimuli and greater motor activity. Thus, reducing amygdala activity by neuromodulation treatments is sufficient to restore a homeostatic brain pattern (Siever, 2008; Blair, 2016; Gouveia et al., 2019).

We observed an overall reduced cortical thickness in refractory patients compared to non-refractory patients. It is not possible to determine, however, the underlying cause of this morphological difference, but it is plausible to assume that the chronic use of high dose mono and poly-pharmacy by the refractory group could result in morphometric brain changes, including alterations in cortical thickness. A further cortical thickness analysis of images acquired before surgery revealed significant differences in the pattern of local cortical thickness between refractory and non-refractory patients in areas related to the control of aggressive behavior, such as the orbitofrontal cortex, anterior and posterior cingulate cortices and medial prefrontal cortex (Siever, 2008; Gouveia et al., 2020). Interestingly, no significant local cortical thickness differences can be found any longer after surgery, which corroborates the theory that the functional neurosurgical procedure causes plastic changes and widespread modifications in brain morphology associated with the restoration of brain equilibrium and concomitant improvement in behavior. These characteristic changes are found in all three patients, suggesting that these changes occur within the first months (3 months is the shortest duration since the procedure in patient 2) and remain stable after.

This study provides the rare opportunity to investigate the mechanisms underlying symptom improvement following amygdala ablation in patients that are refractory to all known treatments, with focus on brain-wide cortical remodeling. Although these data allow true causal inferences to be tested, some limitations are to be noted. Refractory patients treated with amygdala ablation are extremely rare, and studying highly aggressive patients that are not collaborative in clinical settings is a very challenging task; nevertheless, the small sample size prevents a greater generalization of the findings. All patients included here required image acquisition to be performed under sedation; however, these patients present a high risk for respiratory depression and death during sedation as a result of the pharmacological regime used to control aggressive behavior. Thus, non-refractory patients (included in the control group) were scanned only once, and no longitudinal analysis could be performed, limiting our knowledge about possible cortical thickness changes in patients treated only with medication. In this sense, this study does not attempt to compare cortical thickness changes in patients subjected to distinct treatments for aggressive behavior but aims to demonstrate that the pattern of cortical thickness observed in non-refractory patients after amygdala lesion (when patients return to show beneficial responses to pharmacological therapy) is similar to the one found in patients that do respond to medication. It is important to highlight that only ASD patients were included in this study as it has been shown that this patient population presents different brain morphology than healthy controls with greater cortical thickness (Bedford et al., 2020).

This study showed evidence that bilateral amygdala radio-frequency ablation induces profound neuronal plasticity along the neurocircuitry of aggressive behavior resulting in brain morphological changes that could be integral to the behavioral improvement observed. Further studies are necessary to determine the underlying cause of these morphological changes and to better understand and improve treatment options.

## Data Availability Statement

All data generated and analyzed during this study are available from the corresponding author upon reasonable request.

## Ethics Statement

The studies involving human participants were reviewed and approved by the Comissão de Ética para Análise de Projetos de Pesquisa do Hospital das Clinicas da Faculdade de Medicina da Universidade de São Paulo (CAPPesq HC FMUSP). Written informed consent to participate in this study was provided by the participants’ legal guardian/next of kin (CAPPesq #742.331; CAAE #31828014.6.3001.5461).

## Author Contributions

FG, JG, and RCRM designed the work. FG, RCRM, EF, RMCBM, and HB acquired the data. FG, JG, and GD analyzed the data. FG, JG, GD, and MC interpreted the data. FG and JG drafted the manuscript. GD, EF, RCRM, HB, MC, and RMCBM critically revised the manuscript. All authors approved the final version of the manuscript.

## Conflict of Interest

The authors declare that the research was conducted in the absence of any commercial or financial relationships that could be construed as a potential conflict of interest.

## Footnotes

1 https://github.com/CobraLab/minc-bpipe-library

2 http://www.bic.mni.mcgill.ca/ServicesSoftware/CIVET

3 https://github.com/Mouse-Imaging-Centre/RMINC

4 https://www.r-project.org/

## Supplementary Material

Supplementary Figure 1 **(A)** Cortical thickness segmentation in patients treated with amygdala lesion **(I,II)** and in controls **(III,IV)**. **(B)** Magnetic resonance imaging of patients treated with amygdala ablation (Cases 1–3, images I–III, respectively) before and after surgery in axial (top row) and coronal (bottom row) planes.
