# Supplementary figures and images for: Bilateral Amygdala Radio-Frequency Ablation for Refractory Aggressive Behavior Alters Local Cortical Thickness to a Pattern Found in Non-refractory Patients
Source: Front Hum Neurosci. 2021 Jun 9;15:653631. doi: 10.3389/fnhum.2021.653631 (PMC8219880; doi:10.3389/fnhum.2021.653631)

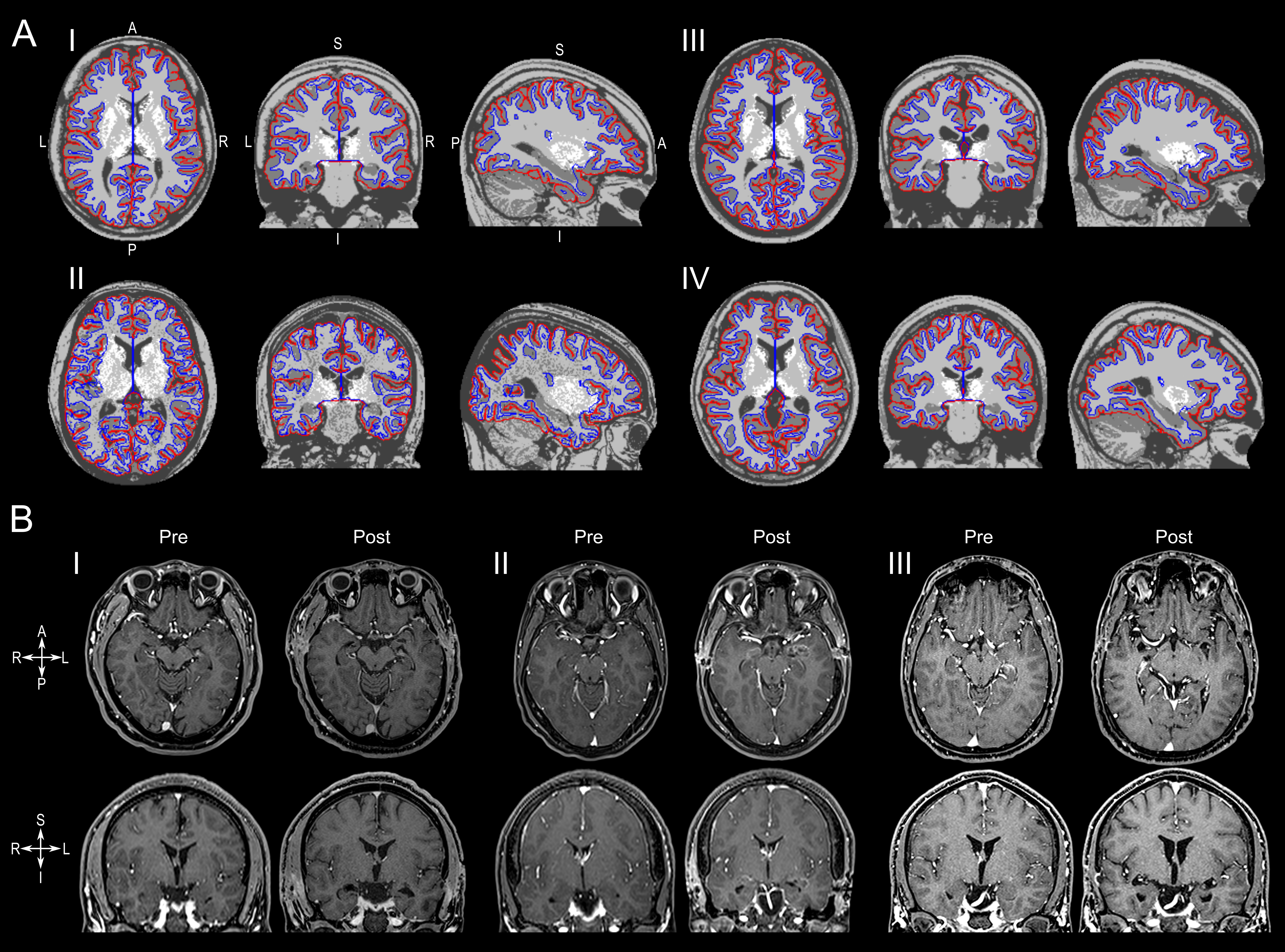

Supplement: Supplementary Figure 1 — (A) Cortical thickness segmentation in patients treated with amygdala lesion (I,II) and in controls (III,IV). (B) Magnetic resonance imaging of patients treated with amygdala ablation (Cases 1–3, images I–III, respectively) before and after surgery in axial (top row) and coronal (bottom row) planes. [file Image_1.PNG]
